# MyHospitalVoice – a digital tool co-created with children and adolescents that captures patient-reported experience measures: a study protocol

**DOI:** 10.1186/s40900-024-00582-2

**Published:** 2024-05-21

**Authors:** Jane Hybschmann, Jette Led Sørensen, Jakob Thestrup, Helle Pappot, Kirsten Arntz Boisen, Thomas Leth Frandsen, Line Klingen Gjærde

**Affiliations:** 1 Mary Elizabeth’s Hospital and Juliane Marie Centre, Copenhagen University Hospital – Rigshospitalet, Blegdamsvej 9, Copenhagen, 2100 Denmark; 2 Department of Clinical Medicine, Faculty of Health and Medical Sciences, University of Copenhagen, Blegdamsvej 3B, Copenhagen, 2200 Denmark; 3 Department of Oncology, Copenhagen University Hospital – Rigshospitalet, Blegdamsvej 9, Copenhagen, 2100 Denmark; 4 Department of Paediatrics and Adolescent Medicine, Center of Adolescent Medicine, Copenhagen University Hospital – Rigshospitalet, Blegdamsvej 9, Copenhagen, 2100 Denmark

**Keywords:** Patient-reported experience measure, Child participation, Adolescent participation, Research involvement, Patient and public involvement, Co-creation, Hospital experience, Quality of healthcare

## Abstract

**Background:**

Children and adolescents have the right to participate in decisions concerning their health and express their views, also regarding hospital experiences. Patient-reported experience measures (PREMs) are valuable tools for systematically incorporating patient voices into healthcare systems. New developments have focused on PREMs for children and adolescents, though they are more commonly used in adults. A recent systematic review mapping their use for children and adolescents indicates a growing interest in this area. However, most PREMs are completed by proxy, in this case parents, so they do not necessarily reflect children’s experiences or align with their rights. Innovation is required to support and engage children and adolescents in responding to these types of questionnaires.

**Methods:**

Collaborating with children and adolescents (4–17 years), the primary aim of this study is to develop and validate the tool MyHospitalVoice containing digital and developmentally appropriate PREMs. The secondary aim is to document and evaluate the approaches used to involve children and adolescents and to assess the impact of their involvement. Based on the European Organisation for Research and Treatment of Cancer framework, we will divide its development and validation into four phases. First, we will discuss PREM items with children and adolescents, who will select and prioritise what they perceive as most important. Second, we will create items targeting different age groups (4–7, 8–12, and 13–17 years) and design a responsive digital interface with child and youth friendly ways of responding to the questionnaires. Third, we will explore how children and adolescents perceive MyHospitalVoice using cognitive interviewing techniques and other age-appropriate methods. Last, we will pilot test MyHospitalVoice to explore patient experiences and response rates. In each phase, children and adolescents will play an active role. We will involve young adults as peer researchers in the project group to ensure that their perspectives are part of the decision-making process.

**Discussion:**

This project will contribute to research on co-creating with children and adolescents and enhance our understanding of their patient experiences. A validated tool like MyHospitalVoice can help improve quality of care by translating the needs and preferences of children and adolescents into clinical practice.

**Supplementary Information:**

The online version contains supplementary material available at 10.1186/s40900-024-00582-2.

## Abstract (significance)

We will collaborate with children and adolescents (4–17 years) to develop a digital tool called MyHospitalVoice. We believe that we can improve the quality of children and adolescents’ healthcare encounters by using MyHospitalVoice to incorporate their needs and preferences into clinical practice. Children and adolescents have the right to be involved in decisions about their health, also when in hospital. Being in hospital can be tough for children and adolescents and adults may not always pay enough attention to what children and adolescents experience. Giving children and adolescents an easy way to share their needs and preferences may protect their rights and ensure that healthcare professionals consider them more. One way to let patients share their hospital experience is by using questionnaires called patient-reported experience measures (PREMs). While similar tools are used for adults, there is a growing recognition of the need for them specifically tailored to the perspectives of children and adolescents. This project will develop a digital tool called MyHospitalVoice, which consists of PREMs designed for and with children and adolescents. We will follow a four-phase process based on established guidelines. We will gather existing PREMs and let children and adolescents decide which questions are most important to them. Next, we will create questions that are suitable for different age groups (4–7, 8–12, and 13–17 years) and design a user-friendly digital interface with playful features to make the questionnaire more engaging. Finally, we will assess how children and adolescents perceive MyHospitalVoice and end by doing a pilot test.

**Supplementary Information:**

The online version contains supplementary material available at 10.1186/s40900-024-00582-2.

## Background

Children and adolescents have a right to participate in matters regarding themselves and their health, just as they have a right to express their own views [1, 2]. These rights also apply to children and adolescents in hospital. Moreover, healthcare systems have a responsibility to facilitate and support these rights. Hospitalisation places children, adolescents, and their families in a particularly vulnerable life situation, where there may be little awareness of the child’s rights or the capacity to prioritise them. Thus, providing them with a tool to make their voices heard represents a way to secure these rights and put them more in focus. Patient-reported experience measures (PREMs) are a valuable method for systematically incorporating the voices and participation of children and adolescents into the healthcare system [3]. PREMs are questionnaires that systematically measure the patient experience, providing patients with the opportunity to express their views on their encounter with the hospital and healthcare professionals [4].

PREMs, which make it possible for hospital administrators and healthcare professionals to gain valuable insights into what is important from the patient perspective, are useful in identifying areas of improvement and in translating patient needs and preferences into actual qualitive improvement projects to promote patient-centred care [5].

Although PREMs are still more commonly used in adult patient populations, the development, validation, and implementation of PREMs for children and adolescents have increased rapidly over the last few years [6, 7]. In 2021, a systematic review on their use for children and adolescents in high-income countries included 39 different PREMs reported in 83 peer-reviewed articles [4]. The review showed that only six of the 39 PREMs had been developed explicitly for completion by children and adolescents, with the remainder completed by parents (as proxies) or not reported. Asking the parents about their child’s experience does not correspond to the rights of the child, with research showing that parental evaluation of their child’s experience may not accurately represent the views and experiences of the child [8, 9].

Since the review’s publication, more action has been taken to represent the voice of children and adolescents. The overall goal of a European project initiated in 2021 entitled, *V*alue *o*f *i*ncluding the *C*hildren’s *E*xperience for improving their right*S* during hospitalization’ (VoiCES) is to develop and implement a joint PREM observatory across European children’s hospitals [10]. Involving several phases and qualitative and quantitative methods, researchers, clinicians, children, adolescents, and families from Finland, Italy, Latvia, and the Netherlands participated in developing and validating the VoiCES PREMs. Consensus has been reached on a final version of (age grouped) PREMs but implementation is in its early stages. No results have been published yet and the questionnaires are not publicly accessible.

The implementation process plays a particularly key role in the feasibility and applicability of PREMs, just as the mode of administration has a prominent role. The VoiCES PREMs will be administered electronically, even though the authors of the aforementioned systematic review found that paper and pencil are still most commonly used [4], for example Wray et al.’s Children and Young People’s PREM (CYP-PREM), which was developed and validated *for* and *with* children aged 8–11 and 12–15 years of age at Great Ormond Street Hospital in London [11]. The CYP-PREMs have been translated and culturally adapted to at least four other languages [12, 13].

The response rate on these paper-and-pencil PREMs in the UK is around 24% [14], which is considered acceptable but there is still room to engage even more children and adolescents in actively participating in the evaluation of their hospital experiences. In a study assessing the implementation of PREMs for children in Canada, McCabe et al. found that researchers, PREM administrators, and healthcare professionals express concerns about paper-and-pencil PREMs because they perceive this mode of administration as a hindering factor in their implementation and utilisation [15]. The authors set out a list of recommendations for integrating PREMs into the paediatric health system. Along with endorsing electronic platforms as preferable, the authors also list short yet flexible measures with multiple forms to reflect various age groups as a priority. They also stress the importance of clinicians knowing that the measures are relevant, reliable, and valid for their intended use. In addition, engaging patients, families, and staff in planning their implementation might also ease the implementation [15].

To accommodate these recommendations and address some of the challenges and gaps in research on PREMs for children and adolescents, we will build on the existing literature, co-create, and validate MyHospitalVoice in collaboration with children and adolescents with hospital experiences. The MyHospitalVoice tool will contain developmentally appropriate questions and response options (questionnaires) for children aged 4 to 7 year, 8 to 12 years, and adolescents aged 13 to 17 years about their hospital experiences and also feature an interactive digital interface that adapts to the age of the user (when they respond to the questionnaires). We intend to engage children and adolescents with hospital experiences not only as informants but also as active collaborators in shaping the project and during methodological considerations when appropriate and meaningful. Previous research shows that children and adolescents can contribute considerably as informants, testers, and to some extent as partners [16, 17]. In accordance with the National Institute for Health and Care Research’s definition of the involvement of children as “research being carried out ‘with’ or ‘by’ rather than ‘to’, ‘about’ or ‘for’ them’” [18], we also consider the engagement of children and adolescents as co-creation, which is defined as “to create jointly” or “to create (something) by working with one or more others” [19].

Together with children and adolescents, our primary aim is to develop and validate MyHospitalVoice, while our secondary aim is to document and evaluate the approaches used to involve children and adolescents and to assess the impact of their involvement on the process and MyHospitalVoice.

## Methods

The development of MyHospitalVoice will involve creating a set of developmentally appropriate items (questions and response options in the questionnaires) and also an interactive digital interface that adapts to the age of the users (this interface is what the child/adolescent sees when filling out the MyHospitalVoice questionnaires).

Based on the European Organisation for Research and Treatment of Cancer’s (EORTC) framework for developing and validating questionnaire modules [20], we will build on existing PREMs for children and adolescents [10–13] and collaborate with children and adolescents to co-create, digitalise, and validate our MyHospitalVoice PREMs.

### Project structure, participants, and recruitment

Involvement of children, adolescents, and young people is central to this project, and the involvement appears on two separate levels: project level and content level (illustrated in Additional file 1).

At the project level, we will involve three adolescents and/or young adults as peer researchers in the project group which means they are involved in planning and conducting of workshops, interpreting and dissemination of results from the workshops, and discuss next steps throughout the study and thus share decision-making power with the researchers. They will be recruited through an established network called the Youth Panel at Copenhagen University Hospital – Rigshospitalet, Denmark [21]. The Youth Panel, which comprises 15 adolescents and young adult patients 15–24 years of age, meets eight times a year to discuss new initiatives to make the hospital more youth friendly. The Youth Panel also engages in training staff and quality improvement projects, in addition to awarding an annual prize to a healthcare professional who puts in a special effort for adolescents and young people. Although the young adults are not part of paediatrics in Denmark (0 to 17 years), we have chosen to involve young adults at the project level both for practical reasons (Youth Panel is already established) and to recognise the potential work burden and complexities of this project. To acknowledge the work burden, the Youth Panel members will be asked to engage for shorter time periods (e.g. 3 months) and then reconsider and potentially switch place with someone else at the end of the period. Those who engage will be paid for the time they invest in the project based on a student assistant salary rate. They decide how much time they invest in the project, but as a starting point they will be invited to: (1) An introductory meeting (about co-conducting research and PREMs), (2) Planning of one workshop (two meetings), (3) Co-facilitate a workshop, and (4) Discuss findings and next steps.

At the content level, we involve children and adolescents from the paediatric population (4 to 17 years, thus not the youngest age group of 0 to 3 years) though workshops. The workshops will be age grouped (4 to 7 years, 8 to 12 years, and 13 to 17 years) and be about either the age-specific questionnaire or age-specific digital interface. For these workshops, we will invite children and adolescents from various hospital departments at Rigshospitalet as well as children and adolescents who are part of the MARYS’ user panel [22], which is being used in the planning, development, and conceptualisation of Rigshospitalet’s new children’s hospital called Mary Elizabeth’s Hospital (MARY). The user panel is used to recruit participants, e.g., for user tests, focus groups, and research purposes. When the panel was established, information about it and how to register was published on social media forums and in posters and flyers at departments for children and adolescents at Rigshospitalet. To date, the user panel contains 127 children and adolescents 0–17 years of age and their or their parent(s)’ contact information. To recruit participants for the MyHospitalVoice project, we will contact them by e-mail using Research Electronic Data Capture (REDCap) [23].

For the workshops, the participant inclusion criteria are 4–17 years of age, having experienced being in a hospital, and being able to speak and understand Danish. In addition, the parents must understand written Danish, English, or another Scandinavian language to be able to provide written consent for their child’s participation. We have not predefined any other criteria for patient characteristics, e.g. gender or ethnicity, thus the recruitment is based on convenience sampling and might not represent marginalised voices. Workshop participants will have their travel costs reimbursed and food and drinks will be provided at the workshops.

### Approaches to document and assess involvement

Standards for patient and public involvement in research and models for participatory design will guide the involvement of children, adolescents, and young adults [24–27]. We will report the involvement according to the Guidance for Reporting Involvement of Patients and the Public Checklist (GRIPP2) [28] (can be found in Additional file 2).

Preston et al. [29], who tested the Patient Engagement Quality Guidance Tool [30] in cases that involved children and adolescents in research, found that it was helpful and informative in terms of systematising reflections on the practicalities and experieces of involving them in research and to identify gaps in practice. Based on their suggestion, we used the tool in the planning stage but in an adapted version that is available in Additional file 1.

**Fig. 1:**
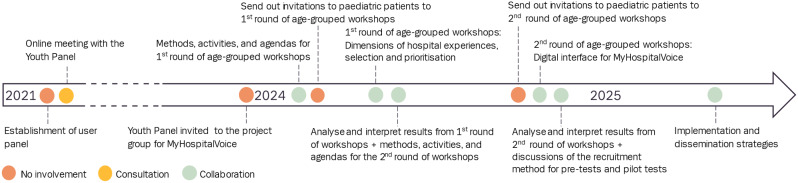
Planned activities that are related to the involvement of children, adolescents, and young people. Preston et al.’s [32] matrix guided the use of wording

Inspired by Dawson et al. [31] we state here our (a priori) planned involvement activities and will then compare them with the actual involvement activities at the end of the project. Our comparisons will be made based on notes and minutes from meetings. For each activity, we will reflect on whether our expectations were met and how the involvement impacted and shaped the project (Fig. 1).

If we find that the degree and frequency of involvement is not feasible and/or meaningful, we will reconsider our initial plans and seek alternative ways to involve children, adolescents, and young adults. We expect our a priori and explicitly documented plan will help guide our evaluation of involvement and aid in defining and describing the facilitators and/or barriers to involvement, regardless of whether our approach is successful or will require an alternative plan. In Additional file 1, we have included a matrix adapted by Preston et at [32] to illustrate our planned degree of involvement throughout the research phases.

The adolescent/young adult peer researchers will be asked to fill out the Patient Engagement in Research Scale (PEIRS-22) to measure the engagement at ‘project level’. The scale has been developed to measure meaningful patient engagement [33] and was recently translated and culturally adapted to Danish [34].

### Phases of the project

**Fig. 2:**
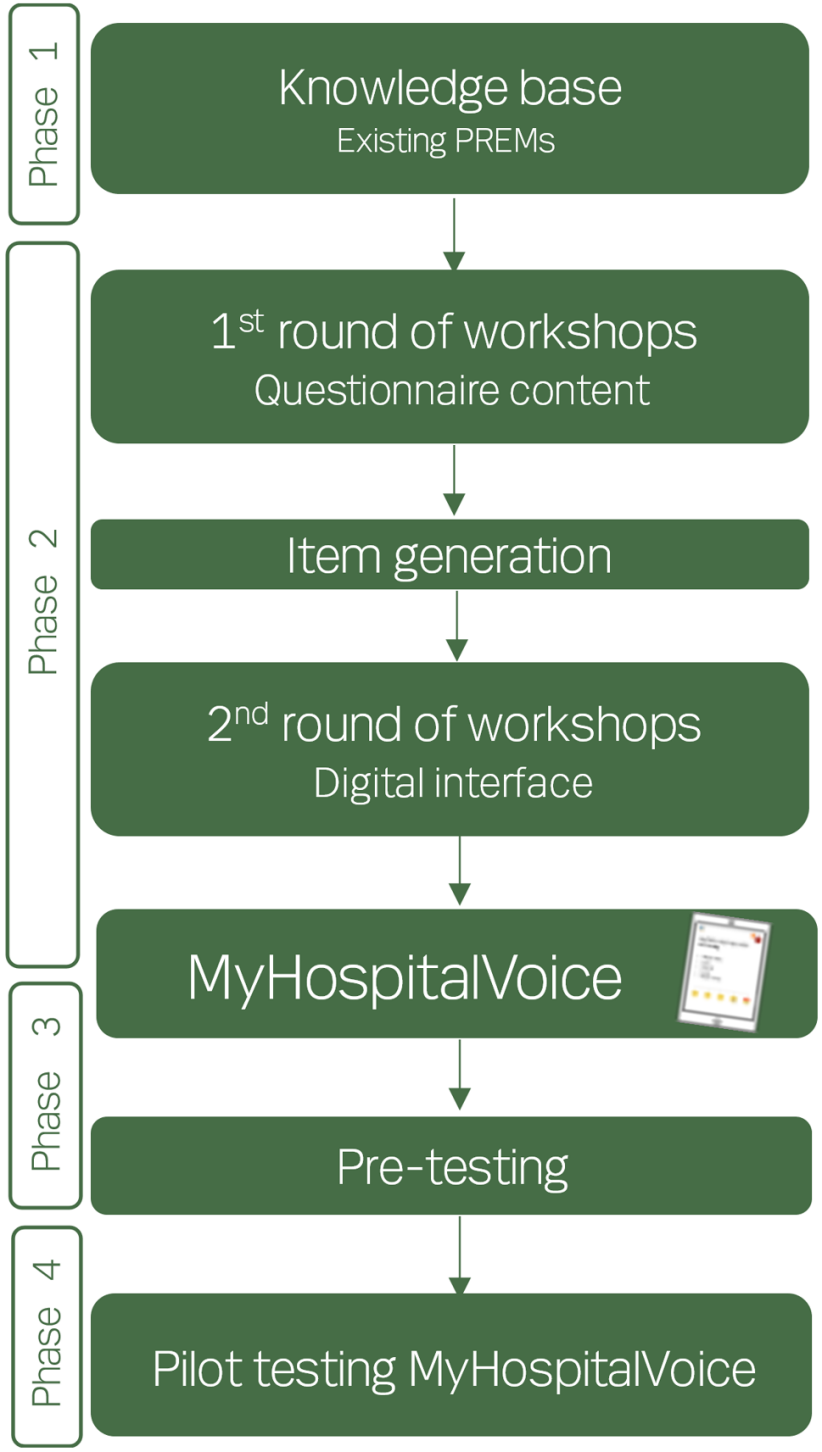
Development and validation of MyHospitalVoice. PREMs: patient-reported experience measures

Based on the EORTC framework, the development and validation process comprises four phases (Fig. 2) [20].

In *phase 1*, the first author (JH) will extract data from existing PREMs for children and adolescents, translate the items into Danish, and categorise ones that relate to the same dimension. Without sharing these dimensions with the adolescent/young adult peer researchers, we will ask them to indicate which dimensions they perceive as important and to suggest new items. After this, researchers and peer researchers will discuss and compare the categories and dimensions based on existing PREMs and the ones the peer researchers provide. We will clarify the translations, rephrase, and choose between redundant items, if necessary. Based on the identified categories and dimensions, the researchers and peer researchers will plan co-creative workshops to be held in phase 2.

In *phase 2*, we will conduct three age grouped workshops to co-create the questionnaire content, using participatory design methods to guide them [24, 35, 36]. The format and content will depend on the age group: 4 to 7 years, 8 to 12 years, and 13 to 17 years with eight to 16 participants at each workshop. For children and adolescents aged 8 to 17 years, parents are allowed to participate, if their child wants them to. For children 4 to 7 years, parents will participate but be encouraged to let their child be in control.

We will start with running workshops for the oldest age group and then progress to the younger ones building on knowledge gained in workshops for older children/adolescents.

JH will be responsible for organising the workshops and act as the primary facilitator, collaborating with the peer researchers. At the workshops and in developmentally appropriate, playful, and creative ways, the participants will be invited to explore hospital experiences and discuss, select, and prioritise which dimensions they perceive as most important in addition to adding new ones. To provide an example, the workshop for adolescents aged 13 to 17 years will consist of: welcome, ice-breaker activities, process exercises about hospital encounters and experiences, and evaluation and sum-up. A table of agenda and draft activities are provided in Additional file 1. The specific activities and exercises have not been decided yet, as they are to be designed in collaboration with young adult peer researchers, but they will be based on the Danish “Handbook for Child Involvement” (*Håndbog for børneinddragelse*) and Participation Works’ “The Toolkit” [26, 27]. During the activities paper, cardboard, scissors, sticky tape, and stickers will be available and participants will be encouraged to draw, write, discuss, and reflect.

Depending on the age group, we will discuss general considerations regarding questionnaire development, for instance introducing texts and type of response options (e.g. yes/no and Likert scales).

During the workshops researchers will take notes and each activity/exercise and its process and outcome will be reflected upon. After the workshops, the resulting materials will be collected and stored according to Danish regulations on data management. Based on workshop exercises, summing up, and the materials produced, JH will make a first draft of the dimensions, preliminary items, and results to present them to the researchers and peer researchers at a work meeting. JH will facilitate the meeting to ensure that the perspectives of both groups are included in the interpretation of data. We will also compare our items to original items from existing PREMs, and related questions will undergo forward-backward translations [37] to ensure comparability and research collaboration with other countries. The process will be iterative but will result in the first set of questionnaires for MyHospitalVoice.

**Fig. 3:**
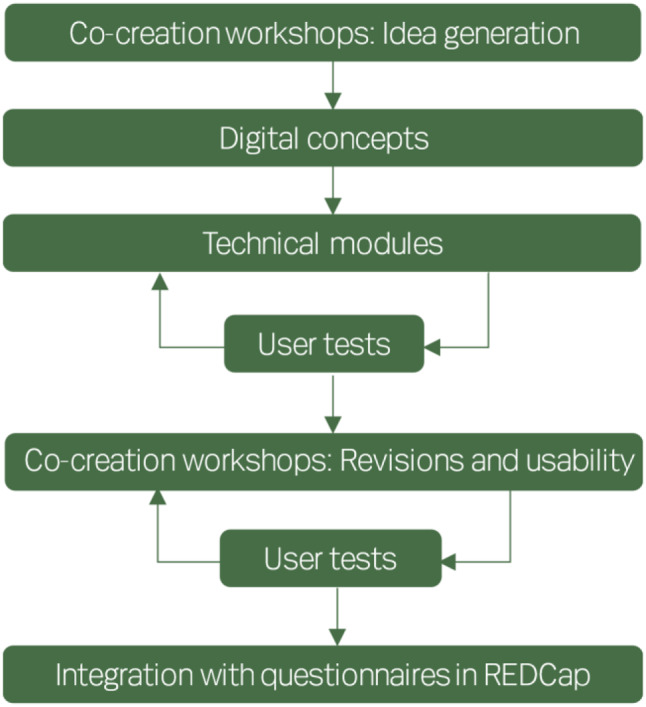
Development of the digital interface for MyHospitalVoice

We will then initiate an iterative creation of the digital interface for MyHospitalVoice (Fig. 3). Again, we will conduct at least three age grouped workshops: 4 to 7 years, 8 to 12 years, and 13 to 17 years with eight to 16 participants at each workshop, starting with the oldest age group. At these workshops, parents are welcome too, but with the child’s view and preferences being the central point.

The children and adolescent for these workshops will be recruited from the same population as the first round of workshops (user panel and paediatric departments), but it is not a requirement to have participated in the first-round workshops about the questionnaire content.

The second-round workshops will engage the children and adolescents in the co-creation of the design of the digital interface. Their ideas will help to incorporate playfulness, storytelling, and perhaps gamification in the development of age-appropriate ways of responding to a questionnaire. Researchers have found that children and adolescents find it easier and more fun to answer questionnaires in an animated application than in a paper questionnaire or orally [38]. Thus, the digital interface for MyHospitalVoice will be age appropriate and incorporate playful aspects. The digital interface will undergo multiple testing and the same or new children/adolescents might be invited to several test sessions.

The questionnaire will be stored in REDCap, and the digital interface will be developed as a responsive website with an application programming interface that pushes data to REDCap when the questionnaires are filled out. Once the digital interface has been developed, we will establish this connection, resulting a beta version of MyHospitalVoice.

In *phase 3*, we will conduct two types of pre-testing. First, the questionnaires will be administered to children and adolescents to identify and solve potential problems in phrasing and/or the sequencing of questions and to identify any missing or redundant items. In the second pre-test, we will conduct cognitive interviews with participants from the target population, children and adolescents with hospital experience, to investigate their understanding of the items in more detail. We will explore how they perceive the items (introductory text, questions, and response options), and the word choice. Cognitive interviewing methods will use the think-aloud method, where subjects are explicitly instructed to think aloud while answering the questionnaire [20]. Studies show that children as young as eight years of age can engage in this process and give meaningful feedback on their understanding of items [39]. After completing the two pre-tests, MyHospitalVoice will undergo refinement and, depending on the extent of the changes required, the pre-testing will be repeated.

In the last phase, *phase 4*, we will pilot test MyHospitalVoice with approximately 200 children and adolescents from in- and outpatient departments at Copenhagen University Hospital – Rigshospitalet. They will also receive a short age-appropriate debriefing questionnaire. There are no formal requirements on sample size in developing and testing a questionnaire, but others have included 10 patients per item, though more with a heterogeneous target population [40]. In the later stages of the project, hospital department leaders will be consulted to help determine which patient populations will take part in pilot testing MyHospitalVoice.

We will evaluate the pilot test(s) by calculating response rates and exploring the factors that either hindered or facilitated implementation. Based on the evaluation, we may incorporate minor modifications. We will explore the psychometric properties of MyHospitalVoice using exploratory factor analysis, confirmatory factor analysis, Cronbach’s alpha, and differential item functioning analysis. We will also perform an exploratory analysis of correlations between experience factors and patient characteristics, e.g. age, disease severity, and prior hospitalisation, in addition to determining the required sample sizes to find associations between experience factors and, e.g. readmission.

### Implementation of MyHospitalVoice

After conducting and evaluating our pilot study, we will implement MyHospitalVoice at Copenhagen University Hospital – Rigshospitalet in paediatric departments, adult departments, and outpatient clinics that admit children and adolescents. The implementation process will start in early phases of the project, so that the MyHospitalVoice tool is designed to fit into existing structures (e.g. the electronic health record system) and clinical practices. To do this, we will have ad hoc involvement of relevant stakeholders (e.g. clinicians, leaders, digitalisation and implementation managers). Implementation will adhere to existing management structures and be coordinated with other relevant bodies.

**Fig. 4:**
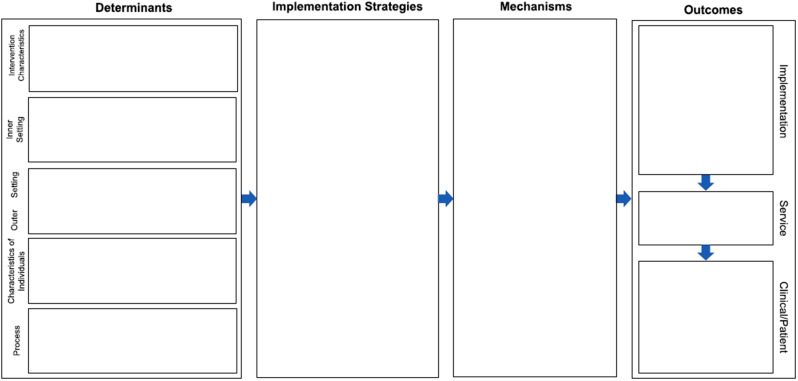
Smith et al.’s implementation research logic model [41]

The Implementation Research Logic Model (IRLM), which will guide the implementation (Fig. 4), is a useful tool for planning, executing, and reporting the implementation of research projects [41]. IRLM incorporates aspects from other well-known implementation frameworks, including the Consolidated Framework for Implementation Research (CFIR) [42]. Factors from CFIR will be included in the first step in the IRLM to help identify the determinants of implementation that can act as either facilitators or barriers to successful implementation. The determinant domains, which include innovation, inner setting, outer settings, individuals, and process, will be identified partly through our pilot study and partly through discussions with stakeholders and staff. Based on the determinants, we will identify and select relevant implementation strategies to facilitate and ease implementation. The mechanisms that relate to how the implementation strategies affect the determinants and their implications for outcomes will be discussed and hypothesised in the research group and with relevant collaborators at Copenhagen University Hospital – Rigshospitalet.

## Discussion

Collecting information on the hospital experiences of children and adolescents can help facilitate translate their needs and preferences into clinical practice and represent a possible way of operationalising patient-centred care. Thus, validated PREMs for children and adolescents can serve as a powerful tool when seeking to improve the quality of care provided to children and adolescents. In contrast to most PREMs for children and adolescents, MyHospitalVoice will provide an interactive digital interface to the questionnaire. A high response rate to the digital questionnaire that we develop will indicate that it is successful and likely increase the impact of MyHospitalVoice. Broad implementation will make rapid, real-time data analysis possible for the benefit of patients, healthcare professionals, and hospital administrators. Moreover, electronic storage of the questionnaire and responses will reduce the work burden for staff and administrators and promote the seamless spread of MyHospitalVoice.

The processes of co-creation during the development of MyHospitalVoice will generate valuable insights into conducting research that is done ‘with’ rather than ‘on’ children and adolescents. We hope to inspire and foster collaboration with other researchers in this area by sharing our methods, knowledge, and experiences of co-creating a digital evaluation tool with children and adolescents.

The results of co-creation will always depend on the people who participate, which is why being aware of and describing the background and prior experiences of researchers, peer researchers, and other participants is essential. Moreover, applying recognised frameworks and guidelines will strengthen our project and help increase the transparency and utilisation of our research.

### Electronic supplementary material

Below is the link to the electronic supplementary material.

Supplementary Material 1

Supplementary Material 2

## Data Availability

No datasets were generated or analysed during the current study.

## Abbreviations

CFIR: Consolidated Framework for Implementation Research
CYP-PREM: Young People’s Patient-Reported Experience Measures
EORTC: European Organisation for Research and Treatment of Cancer
GRIPP2: Guidance for Reporting Involvement of Patients and the Public Checklist 2
IRLM: Implementation Research Logic Model
PEIRS: Patient Engagement in Research Scale
PREMs: Patient-reported experience measures
REDCap: Research Electronic Data Capture
VoiCES: *V*alue *o*f *i*ncluding the *C*hildren’s *E*xperience for improving their right*S* during hospitalization
