# Supplementary material for: MyHospitalVoice – a digital tool co-created with children and adolescents that captures patient-reported experience measures: a study protocol
Source: Res Involv Engagem. 2024 May 21;10:49. doi: 10.1186/s40900-024-00582-2 (PMC11110337; doi:10.1186/s40900-024-00582-2)
Supplement: Supplementary file 1 — Supplementary Material 1 [file 40900_2024_582_MOESM1_ESM.docx]

**Additional file 1: Resources for documentation and reflections on involvement**

**Additional figure 1**. Overview of who participates at project level and at content level in the MyHospitalVoice.


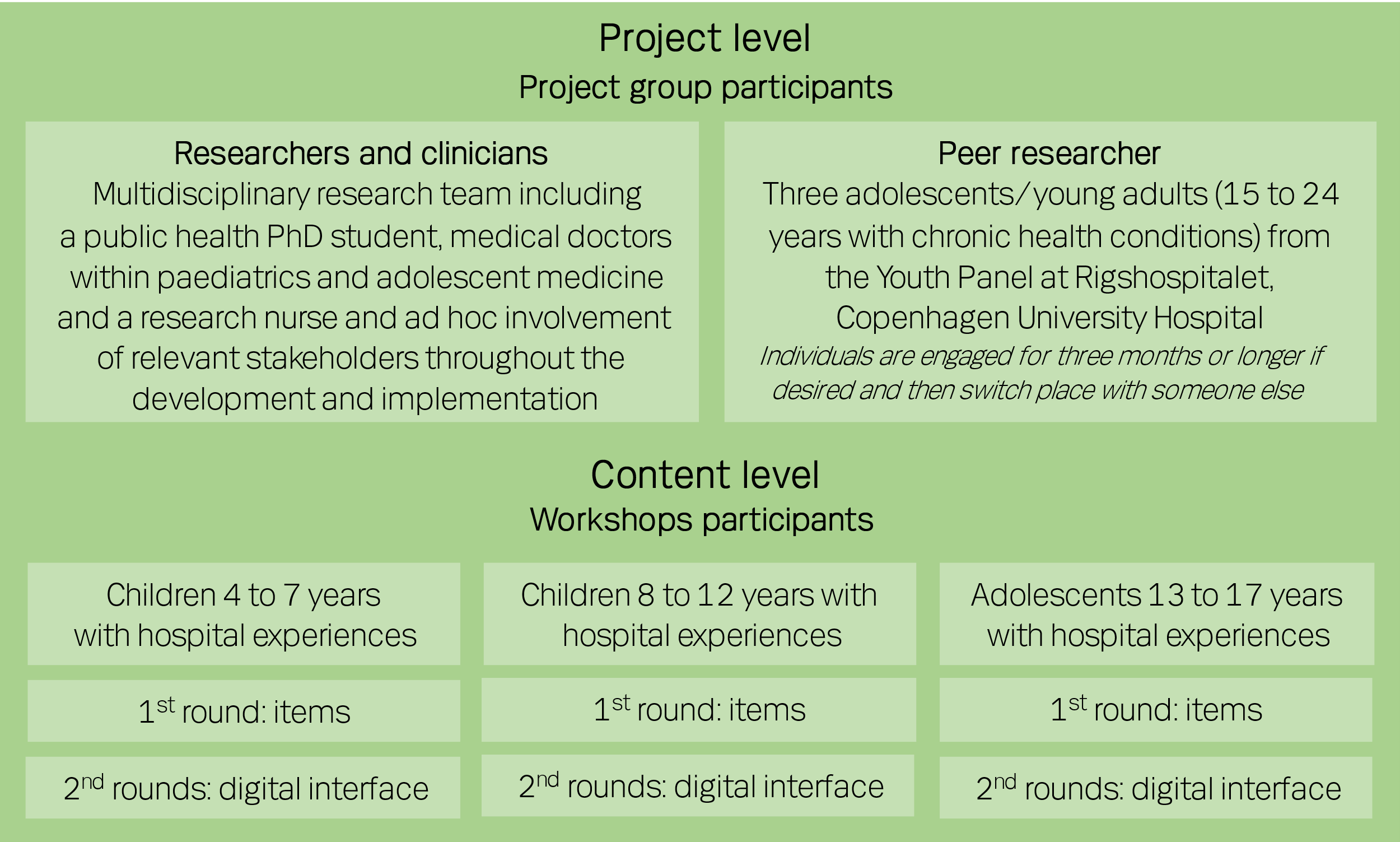


**Additional figure 2**. Planned degree of involvement of children, adolescents, or young adults throughout the research phases. Speech bubbles represent our planned degree of involvement in the different phases.

*
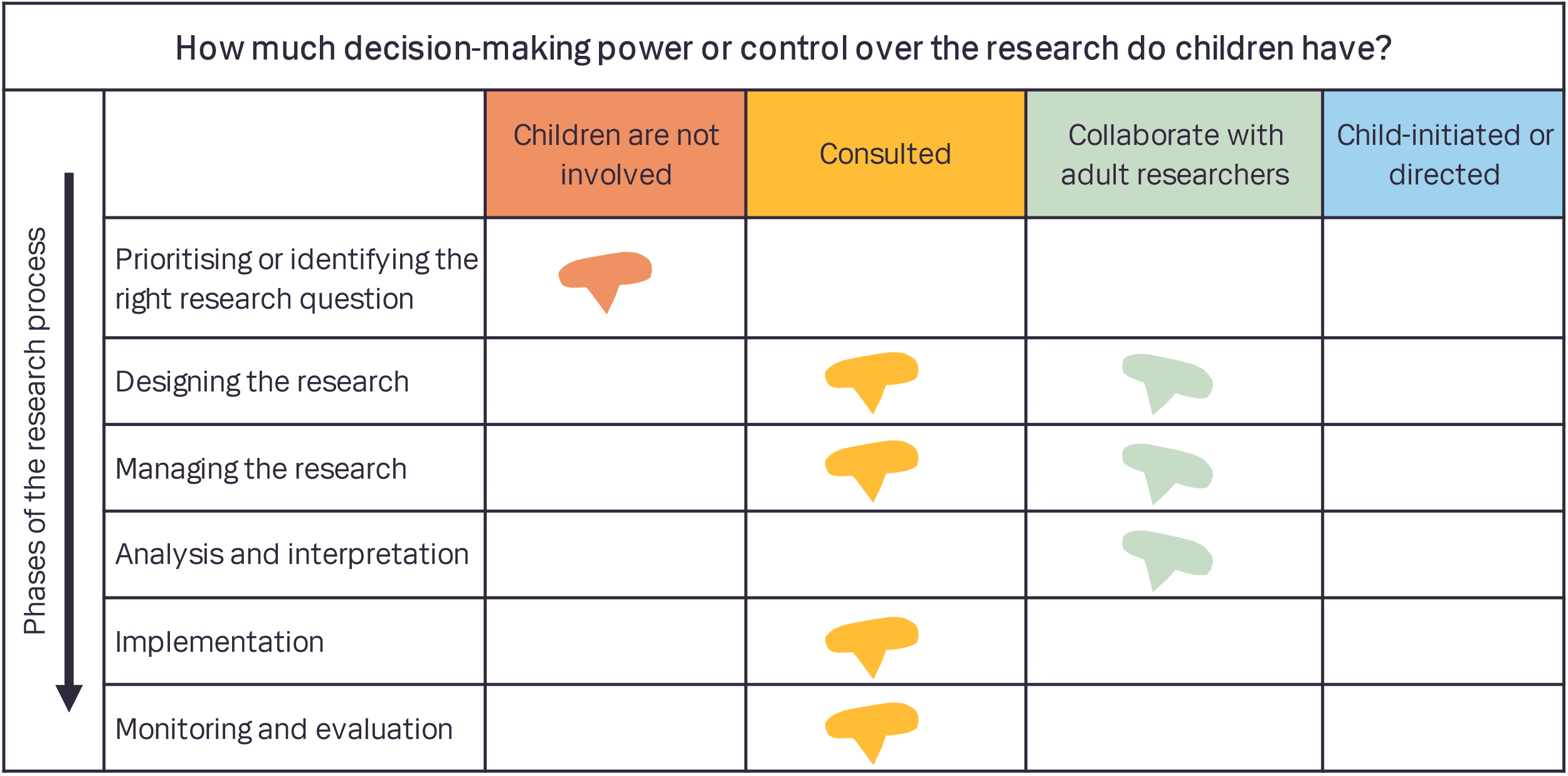
*

**Additional table 1**. Example of structure and preliminary agenda for the workshop for adolescents 13–17 years

| Time | Activity |
| --- | --- |
| Pre-workshop | Send out information about time, place, and purpose |
|  | Welcome: Name tags, brief introduction to project, outline, and purpose of workshop  Water, juice, and fruit available |
|  | Icebreaker: The three things we have in common |
|  | Exercise 1: My hospital encounter |
|  | Short break |
|  | Exercise 2: Hospital experiences: What is that and for whom? |
|  | Pizzas and drinks |
|  | Summing up and evaluation: Our path to the goal |
| Post-workshop | Send out thank yous and short summary of what took place and what will happen next |

**Additional table 2.** Documentation and reflections on involvement of paediatric patients in the development of MyHospitalVoice. *This table is adapted to the project and based on the* *Patient Engagement Quality Guidance Tool and the paper Developing a More Tailored Approach to Patient and Public Involvement with Children and Families in Pediatric Clinical Research: Lessons Learned by Preston J, Nafria B, Ohmer A, Gaillard S, Dicks P, West L, et al.. Ther Innov Regul Sci. 2022;56(6):948-63.*

| **Basic information** | | |
| --- | --- | --- |
|  | The project title | MyHospitalVoice |
|  | Who is involved? | Children and adolescents with hospital experience (as co-creators)  Adolescents and young adults (as peer researchers) |
| **The quality of patient engagement** | | |
| **Shared purpose** *Brief description:* Shared purpose refers to the importance of all stakeholders agreeing on the project’s aims and outcomes before starting the project | | |
|  | What is your stated “shared purpose”? | 1. To develop a digital tool called MyHospitalVoice that consist of patient-reported experience measures for children and adolescents 2. That MyHospitalVoice highlights the importance and value of including the child and adolescent’s voice about hospital experiences to improve the healthcare encounter and secure children’s and adolescents’ rights |
|  | What will you do to achieve this criterion? | The initial plans were shared with the Youth Panel in April 2021. The Youth Panel were interested and wanted to participate in reaching the goal and will be invited as peer researchers. |
|  | How will you confirm with all stakeholders that the purpose is understood, that contributions will influence the original plans, and that disagreements will be addressed? | Openly discuss what the purposes of the project are and revise them accordingly.  On the workshops with children and adolescents, we will use the age-adapted activity “Our way to the goal” where “the goasl” will be discussed (corresponds to the purposes) to reflect on differences and similarities and what we (researcher+peer researcher vs children and adolescents/co-creators) perceive as the purpose. |
|  | Will you plan to review the shared purpose and its understanding among stakeholders? | Yes |
|  | At what time points? | At each meeting with the Youth Panel (peer-researchers) and at each workshop we will briefly summarise our goals and discuss and review the goals |
| **Respect and accessibility**  Brief description: Respect and accessibility refer to (1) respecting each other and respectful interactions within the project to be established among partners, and (2) openness to and inclusion of individuals and communities (to the project) without discrimination | | |
|  | How will you address respect and accessibility in this project? | At the first meeting with the Youth Panel, we will openly discuss plans, expectations, roles, and responsibilities. We will work on and agree upon a shared ‘set of rules’ of how we work together. |
|  | How will you assess with stakeholders that they acknowledge mutual respect, and that access to engagement has been optimised? | As the researchers involved in this project are the ones to meet with the co-researchers and children/adolescents, no other stakeholders have direct contact with children, adolescents, or young adults |
| **Representativeness of stakeholders**  *Brief description*: Representativeness of stakeholders refers to the mix of people involved, which should reflect the needs of the project and the interests of those who may benefit from project outputs | | |
|  | How will you ensure broad, competent, diverse representation of stakeholders? | For practical and ethical reasons, we have chosen to include adolescents/young adults as peer researcher. Children younger than 15 years will be represented through the adolescent/young adult perspective.  Healthcare professionals’ perspective and engagement should also be sought in this project. For now, the plan of involvement only includes children/adolescents.  In later stages, department leaders, managers, and other relevant collaborators will be involved in accordance with a priori plans and protocols |
|  | How will you check that the representation of stakeholders in your project will support achieving project outcomes? | Through the project phases, the research group will discuss if new the current representation of stakeholders is appropriate. The progression of the project will lead to recurrent mapping of relevant stakeholders, who will be contacted accordingly. |
| **Roles and responsibilities**  *Brief description:* Roles and responsibilities refer to the documentation of agreed, and ideally co-created, roles and responsibilities, indicating that all aspects of project needs will be established upfront and revisited regularly | | |
|  | What will you do to achieve clarity and communication as well as regular check-points on roles and responsibilities? | At a first meeting, we will carefully describe roles and responsibilities and make clear that the described roles are negotiable. JH will be responsible for this. Roles and responsibilities will be written in a Danish “script” that will be negotiated and defined in a collaboration between JH and the peer researchers. |
|  | How will you check that all participants understood what their roles and responsibilities are, and what is expected of them? | We will stress the importance of transparency and openness. We will ask how adolescents and young adults perceive their roles and responsibilities. In case role and responsibilities are not aligned with those anticipated by the researchers, we will discuss how to overcome the discrepancies and reach consensus. |
|  | At what frequency will this be checked in? | At each meeting |
| **Capacity and capability for engagement**  *Brief description:* Capacity and capability for engagement refer to (1) capacity as having relevant and dedicated resources from all stakeholders and (2) capabilities for all stakeholders to enable meaningful engagement | | |
|  | What will you do to support building the required capacity and capability for engagement? | Researchers will acquire skills, competencies, and knowledge through relevant courses and through sharing of knowledge with other relevant collaborators. (Center for Patientinddragelse RegionH, Videns- og Kompetencecenter for Brugerinddragelse i Sundhedsvæsenet ViBIS, Research Centre for Patient Involvement ResCenPI network.)  Peer researchers (who have been involved in other projects) will be trained according to the methods we decide on during the project. They will get an introduction to general research principles, questionnaire development etc. |
|  | How will you check that all stakeholders have what they need to contribute effectively and meaningfully? | Openly discuss if our skills and competencies align with the proposed methods. Seek ways to gain new competencies or collaborations or what mapping of needs tells us. |
| **Transparency in communication and documentation**  *Brief description:* Transparency in communication and documentation refers to the establishment of a communications plan and ongoing project documentation that can be shared with stakeholders. Communication among stakeholders must be open, honest, and complete | | |
|  | What will you do to achieve and implement processes for timely communication and updated documentation throughout the project? | In the protocol article, figure 1-4 depicts which tools we will use for documentation and communication about the processes of involvement.  Language style will be adapted according to context.  JH will be responsible for the communication with the peer researchers and initial contacts with new collaborators, but the whole research group will be involved in the planning and progression from development to implementation. |
|  | How will you validate that your communication and documentation plans are useful and appropriately implemented? | The plans will be discussed and reviewed in the multi professional research group and further by relevant collaborators including department leaders, managers, healthcare staff, and bodies supporting implementation processes. |
| **Continuity and sustainability**  *Brief description:* Continuity and sustainability refers to the smooth progression of the project and efforts to maintain relationships with stakeholders beyond a single project | | |
|  | What will you do to achieve this criterion? | Continuity will be secured through JH as principal investigator and involved in all aspects of the project and all contacts to collaborators/stakeholders. Feedback and status on the project and progression will be shared with collaborators continuously. Milestones are specified in a document that can be accessed upon request to JH. These milestones will be assessed continuously. |
|  | How will you gather feedback on what you have done? | The research group will meet once quarterly and assess if the planning, methods, and milestones are appropriate and achievable. |
|  | How will you check that your planning to secure continuity and sustainability is appropriate also for the stakeholders you’ve involved in the project? | This will be discussed openly with collaborators/stakeholders throughout the projected and be adapted accordingly. |
| **Results and outcomes** | | |
|  | Expected impact for patients (children and adolescents) | MyHospitalVoice will provide children and adolescents with a tool to share their experience of the hospital encounter. This will lead to improvement projects based on the feedback gathered by the tool and an improvement in hospital experiences. Further, children and adolescents will be empowered to share their views and preferences, and this will secure their rights in hospitals. |
|  | Expected impact for healthcare professionals | MyHospitalVoice will provide healthcare professionals with unique insights into what are important for different age groups of children/adolescents when they are in the hospital. This will lead to better tailoring of needs and provide an opportunity for mapping the needs of children/adolescents to healthcare professionals’ skills and competencies.  Further, MyHospitalVoice will be a tool for real time data on hospital experiences that will foster sharing of knowledge and skills between healthcare professionals and hospital departments. |
|  | Expected impact for hospital administrators | MyHospitalVoice will provide hospital administrators with tool to incorporate the child/adolescent voice into planning of healthcare services. Further, the continuous collection of hospital experiences provides hospital administrators with a valuable tool to evaluate both local initiatives and larger projects. |
|  | Expected impact for researchers | The development and validation of MyHospitalVoice will result in several scientific publications   1. Protocol article for the development, validation, and involvement processes 2. Study on children/adolescents as co-creators in defining hospital experiences for Danish children and adolescents 3. Study on children and adolescents as co-creators in the design of an age-appropriate digital interface for MyHospitalVoice 4. Study on the development and evaluation of MyHospitalVoice including the mapping of children’s and adolescents’ hospital experience in a large tertiary Danish hospital   These studies will be published open access and be presented at relevant conferences to foster sharing of knowledge and research collaborations. The knowledge generated will enhance the emerging field of co-creating with children and adolescents with hospital experiences or in a healthcare setting. |
